# Oncocytoma of Oral Cavity Mimicking as Jaw Tumor

**DOI:** 10.1155/2014/315058

**Published:** 2014-11-02

**Authors:** Aloke Bose Majumdar, Shib Shankar Paul, Gautam Sarker, Souradeep Ray

**Affiliations:** ^1^Barasat Cancer Research & Welfare Centre, West Bengal, India; ^2^Department of Otorhinolaryngology & Head Neck Surgery, M.G.M. Medical College & L.S.K. Hospital, Kishanganj, Bihar 855107, India; ^3^Department of Community Medicine, M.G.M. Medical College & L.S.K. Hospital, Kishanganj, Bihar 855107, India; ^4^M.G.M. Medical College & L.S.K. Hospital, Kishanganj 855107, India; ^5^College of Medicine & Sagore Dutta Hospital, Kamarhati, West Bengal, India

## Abstract

Oncocytoma of major salivary gland is a fairly common benign tumour encountered, but its occurrence in oral minor salivary gland is a rare entity. Here we report a case of a giant minor salivary gland oncocytoma mimicking a jaw tumour which was successfully excised along with a review of literature.

## 1. Introduction

Oncocytoma is a benign tumor arising from the oncocytes which line the duct of salivary glands and are fairly common in major salivary glands. They occur very rarely in the minor salivary glands. Histologically, World Health Organization (WHO) (1991) classified them into three distinct types: oncocytosis, oncocytoma, and oncocytic carcinoma. Oncocytoma is also known by oxyphilic adenoma and oxyphilic granular cell adenoma. Oncocytoma is a rare benign salivary gland neoplasm composed of large epithelial cells with characteristic bright eosinophilic granular cytoplasm (oncocytic cells). It accounts for approximately 0.4–1% of all salivary gland neoplasms, occurring primarily in parotid glands, with only a small percentage occurring in minor salivary glands of palate, tonsillar fossae, larynx, nasal cavity, maxillary sinus, and the lacrimal gland. It occurs primarily in persons older than 50 years of age. Only 17 cases of histologically verified oncocytoma of an intraoral minor salivary gland are reported in literature with the 18th case being reported by Palakshappa et al. in 2014 [1]. Here we report a case of oncocytoma, arising from intraoral minor salivary glands, in a 53-year-old female patient.

## 2. Case Report

A 53-year-old female patient presented to the outpatient department of E.N.T. at the Barasat Cancer Hospital, Kolkata, India, with complaints of a slowly growing large swelling in the left lower jaw for a duration of 14 months. There was slight difficulty in chewing and swallowing due to the size of the tumor. On inspection a firm smooth lobulated mass was seen arising from the lingual aspect of the left side of lower jaw of 5 cm by 2 cm in size extending from canine to the premolar teeth occupying the edentulous portion of left half of mandible and adjacent floor of mouth. The mass was painless and nontender on palpation. The teeth on the left half of mandible were loose and patient was unable to approximate the jaws completely; hence, he could not chew or swallow effectively.

The rest of oral cavity oropharynx and laryngopharynx were normal on examination. There were no palpable cervical lymph nodes. Other than diabetes patient had no significant systemic ailments. Other than elevated blood sugar the routine haemogram, serological studies, X-ray of chest, and ECG were within normal limits. An orthopantomogram of the jaw showed a lobulated swelling in the left side of floor of mouth. The hyperglycemia was controlled with insulin therapy on admission of the patient. A punch biopsy from the mass done elsewhere was reported as oncocytoma.

The patient was planned for surgery. By a left lip splitting cervical collar line incision a flap was elevated and tumor was exposed. The tumor was attached to the inner (lingual) aspect of the left half of mandible encroaching upon the edentulous part of the mandible. The tumor was excised using electrosurgical instrument along with a marginal mandibulectomy of the involved part of the mandible. The mucosal flaps were elevated from the floor of mouth and buccal mucosa was released to bury the mandibular defect. The wound was closed in layers over corrugated rubber drain. The patient was kept on broad spectrum antibiotics, anti-inflammatory agents, and insulin on a sliding scale along with antiseptic mouth washes. The postoperative period was uneventful. Patient recovered well and was discharged from hospital 10 days after the removal of sutures.

## 3. Discussion 

**Figure 1:**
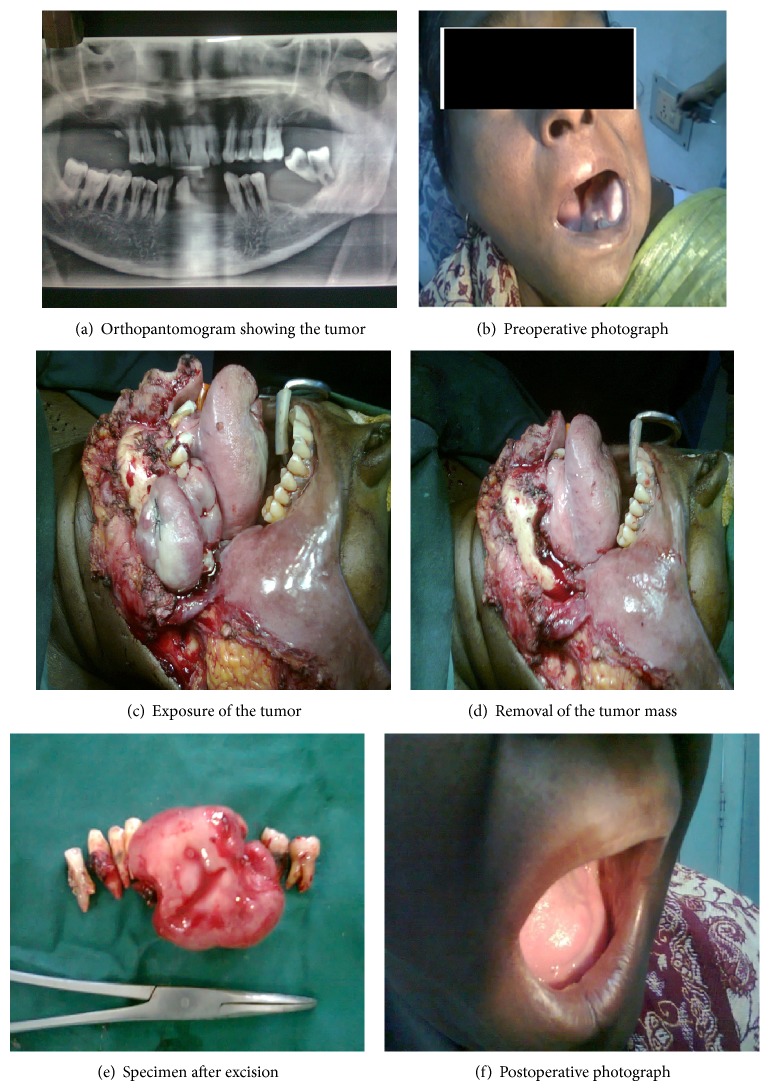
Pictures of oncocytoma.

**Figure 2:**
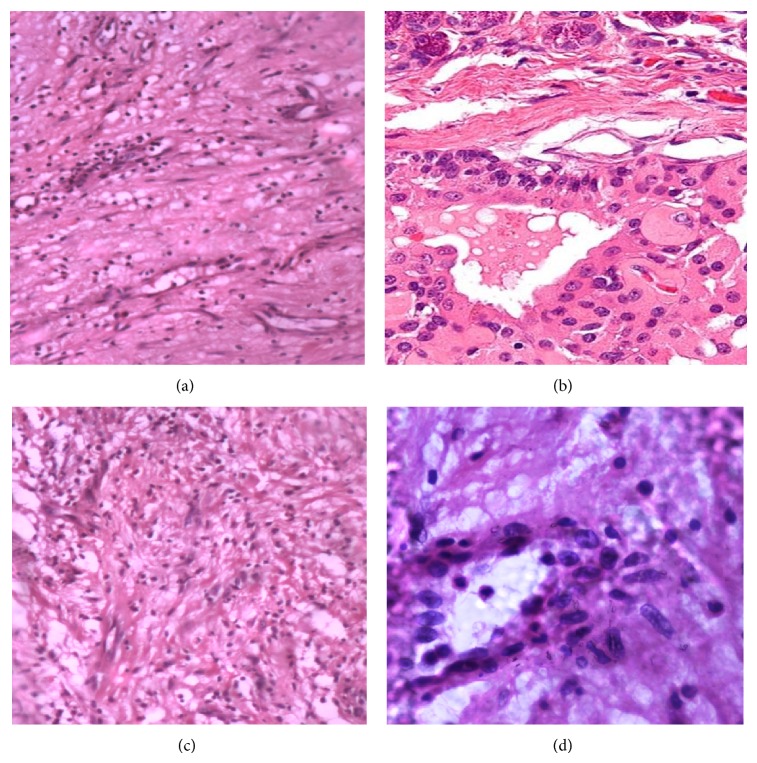
Histopathological pictures of the resected specimen.

Oncocytoma is a rare benign tumor of the salivary gland representing not more than 1% of salivary tumors. It is composed of large epithelial cells, the oncocytes, which are predominantly found, in senior adults, being more prevalent in the eighth decade of life. It is located mainly in larger salivary glands especially in parotid glands [2]. Among oncocytic major salivary gland tumors, 84% occur in the parotid (male to female ratio 1 : 1) and the remainder arises in the submandibular gland. Minor salivary gland sites include the lower lip, palate, pharynx, and buccal mucosa [3]. The tumor usually presents as a solid mass, painless, of slow growth, and rarely it is larger than 4 cm of diameter (Figure 1). There are few reports in literature on minor salivary glands neoplasias. Camara et al. [4] reported 1 case of oral minor salivary gland oncocytoma. The lesion was initially clinically diagnosed as fibroma. On excisional biopsy it was diagnosed as oncocytoma of minor salivary gland. Hamperl is considered to be the “Father of Oncocytes.” He designated “Oncocyte” (from Greek onkosthai—swollen and cytos—cell) as a special type of epithelial cell characterized by a larger than the original cell, with a mitochondria rich dense cytoplasm containing acidophilic granules (Hamperl H). The diagnosis can be confirmed by both light and electron microscopic identification of mitochondrial differentiation [5, 6]. Oncocytic cells in salivary glands can be categorized as oncocytic metaplasia (oncocytosis), nodular oncocytic hyperplasia, and oncocytoma. Brandwein and Huvos [7] defined oncocytoma as a single nodular mass with monotonous appearance and nodular oncocytic hyperplasia as two or more distinct tumor nodules. They are less organized and circumscribed than oncocytoma as per Hartwick and Batsakis. Out of twenty-one parotid oncocytic neoplasms identified, oncocytoma was the most frequent morphology (62%), followed by oncocytosis (28.5%) and oncocytic carcinoma (9.5%). One specimen displayed synchronous oncocytic morphologies (oncocytoma, oncocytosis, and oncocytic metaplasia). One oncocytoma specimen displayed the mtDNA C-tract alteration. Oncocytic neoplasia of the parotid gland is a rare form of salivary gland disease with obscure etiology. The presence of multiple oncocytic morphologies in a single specimen is suggestive of transition between forms (Figure 2). Although oncocytic tumorigenesis secondary to acquired mitochondrial dysfunction is a plausible mechanism, few of these tumors actually harbor mtDNA alterations within the control region [8]. Malignant extra parotid minor salivary gland tumours as compared to parotid malignant oncocytomas present aggressively with multiple cervical lymph node metastasis and cellular ultrastructure had cells filled with mitochondria [9]. Oncocytic adenocarcinoma of parotid gland is more common in the 6th decade of life and later, and those less than 2 cm in size at the initial surgical procedure showed better prognosis [10]. Oncocytoma of the parotid and submandibular salivary glands are rare benign tumours which are even more rare in minor salivary glands (Table 2). Oncocytic metaplasia is far more common than oncocytoma itself. Oncocytomas can be induced in kidneys of rats by the use of N- nitrosomorpholine which induces oncocytic hyperplasia [11]. Yih et al. [12] in their series of 213 minor salivary gland tumours reported pleomorphic adenoma was the most common benign tumor (93 of 119). Canalicular adenoma was the second most common benign one (25 of 119) while oncocytoma was a rare entity. Pires et al. [13] in their review of intraoral minor salivary gland tumours, a clinicopathological study of 546 cases, cited oncocytoma as a rare tumour. Till date, the PubMed and the Medline search and also to the best of our knowledge, has revealed only 17 reported cases of histologically verified oncocytoma of an intraoral minor salivary gland. The 18th case is the first case to be reported in the retromolar region. Their sex, age, and site distribution, together with the current case, are summarized in Table 1. To the best of our knowledge, probably our case will be the 19th case reported as intraoral minor salivary gland oncocytoma.

In our case we encountered a slowly growing large swelling in the left lower jaw for a duration of 14 months. Following all preoperative evaluation, the patient underwent surgery. The excision biopsy was sent for histopathological examination that turned out to be “oncocytoma of minor salivary glands.” Patient is doing well postoperatively and is on follow-up for the last one year with no evidence of recurrence. This case is being reported due to its rarity and unusually large size of presentation.

## Figures and Tables

**Table 1:** Reported cases of minor salivary gland oncocytomas.

Author	Age/sex	Site
Ahlbom [14]	59/F	Hard palate
Miyoshi et al. [15]	53/F	Hard palate
Sato and Watanabe [16]	23/F	Hard palate
Jalisi [17]	42/F	Oropharynx
Crocker et al. [18]	77/F	Palate
Hung [19]	27/M	Hard palate
Matsuda et al. [20]	53/F	Buccal mucosa
Kohno [21]	59/M	Hard palate
Hayashi et al. [22]	62/M	Hard palate
Hayashi et al. [22]	64/M	Soft palate
Regezi et al. [23]	63/F	Buccal mucosa
Chau and Radden [24]	58/M	Buccal mucosa
Damm et al. [25]	73/F	Buccal mucosa
Kochhar et al. [26]	45/F	Hard palate
Kanazawa et al. [27]	32/F	Buccal mucosa
Camara et al. [4]	71/M	Jugal (buccal) mucosa
Yilmaz et al. [28]	72/M	Maxillary posterior alveolus
Palakshappa et al. [1]	32/F	Retromolar area
Present case	53/F	Inner (lingual) aspect of the left half of mandible

F: female; M: male.

**Table 2:** 

Differential diagnosis of oncocytoma
(i) Adenoid cystic carcinoma
(ii) Pleomorphic adenoma
(iii) Adamantinoma
(iv) Chondroma
(v) Warthin's tumor
(vi) Oncocytic carcinoma
